# Prognostic Value of the Prognostic Nutritional Index in Patients with Locally Advanced Bladder Cancer Receiving Perioperative Chemotherapy: A Multicenter Real-World Study

**DOI:** 10.3390/medicina62050992

**Published:** 2026-05-19

**Authors:** Anıl Karakayalı, Mustafa Seyyar, Pervin Can Şancı, Elif Şahin, Berkan Karabuğa, Öztürk Ateş, Burcu Bacak, Meltem Baykara, Görkem Turhan, Hikmet Akar, Ferhat Ekinci, Melek Karakurt Eryılmaz, Berkay Yeşilyurt, Sinem Akbaş, Ali Kalem, Mesut Yılmaz, Ece Demirdelen, Semra Taş, Oğuzhan Yıldız, Özgür Tanrıverdi, Nadiye Sever, Devrim Çabuk, Umut Kefeli, Kazım Uygun

**Affiliations:** 1 Department of Medical Oncology Clinic, Faculty of Medicine, Kocaeli City Hospital, İzmit 41060, Turkey; 2 Department of Medical Oncology, Gaziantep City Hospital, Gaziantep 27470, Turkey; mustafaseyyar27@hotmail.com; 3 Department of Medical Oncology, İzmir Çiğli Training and Research Hospital, İzmir 35620, Turkey; pervin.cansanci@saglik.gov.tr; 4 Department of Medical Oncology, Dr. Abdurrahman Yurtaslan Ankara Oncology Research and Training Hospital, Ankara 06200, Turkey; drbkarabuga@gmail.com (B.K.); dr.ozturkates@gmail.com (Ö.A.); 5 Department of Medical Oncology, Afyonkarahisar Health Sciences University, Kocaeli 03030, Turkey; burcubacak15@gmail.com (B.B.); meltembaykara@yahoo.com (M.B.); 6 Department of Medical Oncology, Karadeniz Technical University Farabi Hospital, Trabzon 61080, Turkey; gorkemturhan@gmail.com; 7 Department of Medical Oncology, Manisa Celal Bayar University Hospital, Manisa 45030, Turkey; hikmetakarr@gmail.com (H.A.); drferhatekinci@hotmail.com (F.E.); 8 Department of Medical Oncology, Necmettin Erbakan University School of Medicine, Konya 42080, Turkey; drangelkarakurt@hotmail.com (M.K.E.); dr.oguzhan@outlook.com (O.Y.); 9 Department of Medical Oncology, Ankara Bilkent City Hospital, Ankara 06800, Turkey; berkay.byi@gmail.com; 10 Department of Medical Oncology, Koç University Hospital, İstanbul 34010, Turkey; sinem_kocak@yahoo.com; 11 Department of Medical Oncology, Faculty of Medicine, Gaziantep University, Gaziantep 27310, Turkey; kalemali88@gmail.com; 12 Department of Medical Oncology, Sakarya Training and Research Hospital, 54100 Sakarya, Turkey; mstsnm08@gmail.com; 13 Department of Medical Oncology, Faculty of Medicine, Trakya University, Edirne 22130, Turkey; demirdelen_ece@hotmail.com; 14 Department of Medical Oncology, Faculty of Medicine, Pamukkale University, Denizli 20070, Turkey; semratasdr@gmail.com; 15 Department of Medical Oncology, Faculty of Medicine, Muğla Sıtkı Koçman University, Muğla 48000, Turkey; dr.ozgur.tanriverdi@gmail.com; 16 Department of Medical Oncology, Haydarpaşa Numune Training and Research Hospital, İstanbul 34668, Turkey; dr.nadya@hotmail.com; 17 Department of Medical Oncology, Faculty of Medicine, Kocaeli University, Kocaeli 41001, Turkey; devrim.cabuk@kocaeli.edu.tr (D.Ç.); umut.kefeli@kocaeli.edu.tr (U.K.); kazim.uygun@kocaeli.edu.tr (K.U.)

**Keywords:** bladder cancer, neoadjuvant chemotherapy, adjuvant chemotherapy, prognostic nutritional index, overall survival, progression-free survival

## Abstract

*Background and Objectives*: Neoadjuvant chemotherapy (NAC) followed by radical cystectomy is the standard of care for eligible patients with locally advanced bladder cancer (LABC). However, adjuvant chemotherapy (AC) remains widely used in real-world practice. Host-related inflammatory and nutritional biomarkers may also influence survival outcomes. This study aimed to compare survival outcomes between NAC and AC and to identify independent prognostic factors for overall survival (OS) and progression-free survival (PFS), with particular emphasis on the Prognostic Nutritional Index (PNI). *Methods*: This multicenter retrospective study included 262 patients with locally advanced bladder cancer. The median age was 66 years, and 84% of patients were male. Patients were treated with neoadjuvant chemotherapy followed by radical cystectomy or adjuvant chemotherapy after surgery between August 2021 and March 2025. The Prognostic Nutritional Index (PNI) was calculated using pretreatment laboratory values. ROC analysis was used to determine the optimal PNI cut-off for predicting mortality, and the derived threshold (49.97) was applied for stratification in all survival analyses. Survival outcomes were evaluated using the Kaplan–Meier method and compared using the log-rank test. Multivariate Cox proportional hazards regression was used to identify independent prognostic factors. *Results*: Among 262 patients, 138 (52.7%) received NAC, and 124 (47.3%) received AC. Median follow-up was 33.6 months (95% CI: 29.4–37.8). No statistically significant differences in OS (*p* = 0.388) or PFS (*p* = 0.499) were observed between treatment groups. In univariate analyses, nodal stage, pathological complete response (pCR), and PNI were significantly associated with both OS and PFS. In multivariate analysis, low PNI (≤49.97) remained an independent predictor of mortality (HR 1.78, 95% CI 1.04–3.38; *p* = 0.044), while N3 nodal stage independently predicted disease progression (HR 5.92, 95% CI 1.06–32.84; *p* = 0.042). *Conclusions*: In this multicenter real-world cohort, nodal stage and systemic inflammatory-nutritional status were key determinants of prognosis in patients with locally advanced bladder cancer receiving perioperative chemotherapy. PNI emerged as an independent predictor of overall survival, suggesting that host-related biomarkers may improve prognostic stratification beyond traditional clinicopathological factors.

## 1. Introduction

Bladder cancer is one of the most prevalent malignancies of the urinary tract, ranking ninth in global cancer incidence and thirteenth in cancer-related mortality [1]. It predominantly affects men and older individuals with comorbid conditions. Approximately 20–30% of patients present with muscle-invasive disease at diagnosis, and despite curative-intent surgery, nearly half develop recurrence within five years [2].

Radical cystectomy with pelvic lymph node dissection remains the cornerstone of treatment for muscle-invasive and locally advanced bladder cancer. However, systemic chemotherapy is integral to improving survival outcomes. Cisplatin-based neoadjuvant chemotherapy (NAC) has demonstrated a consistent survival benefit in randomized trials and meta-analyses, with an absolute improvement of approximately 5–8% in five-year overall survival compared with surgery alone [3,4]. Accordingly, NAC followed by radical cystectomy is currently recommended as the standard of care for cisplatin-eligible patients [5].

Despite this evidence, a substantial proportion of patients are ineligible for NAC due to impaired renal function, poor performance status, or significant comorbidities. In such cases, adjuvant chemotherapy (AC) is frequently administered following surgery when adverse pathological features are present. However, the level of evidence supporting AC remains lower than that for NAC, and real-world treatment strategies continue to vary considerably across institutions [6,7]. Several retrospective analyses and population-based studies have reported comparable survival outcomes between NAC and AC strategies after adjustment for clinical characteristics, suggesting that the sequence of perioperative chemotherapy may be less critical than the administration of chemotherapy itself [7,8].

Beyond treatment sequencing, identifying reliable prognostic biomarkers is essential for optimizing patient management. Among established prognostic factors, pathological complete response (pCR) following NAC has consistently been associated with markedly improved survival, and its attainment is increasingly considered a meaningful surrogate endpoint in bladder cancer [9,10]. Nodal stage also remains one of the most powerful determinants of prognosis, with lymph node involvement strongly associated with increased recurrence risk and decreased survival following radical cystectomy [11,12].

In addition to traditional clinicopathological factors, attention has increasingly focused on systemic inflammatory and nutritional biomarkers as prognostic indicators in oncology. Cancer-related systemic inflammation promotes tumor progression through mechanisms including immune suppression, angiogenesis, and cytokine-mediated modulation of the tumor microenvironment [13,14]. Among the proposed biomarkers, the Prognostic Nutritional Index (PNI)—calculated from serum albumin and peripheral lymphocyte count—reflects both nutritional status and immune competence. Low PNI has been associated with poor outcomes in various solid tumors, including gastrointestinal, hepatobiliary, and lung cancers [15]. Emerging evidence suggests a similar prognostic role in urothelial malignancies; however, data specific to locally advanced bladder cancer patients receiving perioperative chemotherapy remain limited [16,17].

This multicentric study aimed to compare survival outcomes between patients treated with NAC and AC and to identify independent clinicopathological and inflammatory prognostic factors for overall survival and progression-free survival, with particular emphasis on the prognostic value of PNI.

## 2. Materials and Methods

### 2.1. Study Design and Population

This multicenter retrospective cohort study included 262 patients diagnosed with locally advanced bladder cancer between August 2021 and March 2025. Patients were retrospectively identified from institutional databases across participating centers, and all consecutive eligible patients meeting the inclusion criteria during the study period were included to minimize selection bias. Patients were enrolled from 19 centers across 16 cities in Turkey.

Inclusion criteria were as follows: age ≥ 18 years; histologically confirmed urothelial carcinoma; clinical stage ≥ T2 and/or node-positive disease; and treatment with NAC followed by radical cystectomy, or AC following surgery. All radical cystectomy procedures were performed using a conventional open surgical approach across all participating centers.

Patients with incomplete clinical or follow-up data, non-urothelial histology, or those who did not undergo radical cystectomy were excluded from the analysis.

The study was conducted in accordance with the Declaration of Helsinki and approved by the Non-Interventional Clinical Research Ethics Committee of Kocaeli University Faculty of Medicine.

### 2.2. Data Collection

Baseline demographic and clinicopathological variables were collected from medical records. Collected variables included age, sex, ECOG performance status, smoking history, comorbidities, histological subtype, tumor grade, clinical T and N stage, number of removed and positive lymph nodes, treatment characteristics, pathological response, recurrence, Prognostic Nutritional Index (PNI), and survival outcomes. Overall survival (OS) was defined as the time from diagnosis to death from any cause or last follow-up. Progression-free survival (PFS) was defined as the time from diagnosis to disease progression, recurrence, or death.

### 2.3. PNI Calculation

PNI was calculated from pretreatment laboratory values using the following formula:

PNI = Albumin (g/L) + 5 × lymphocyte count (10^9^/L).

**Figure 1:**
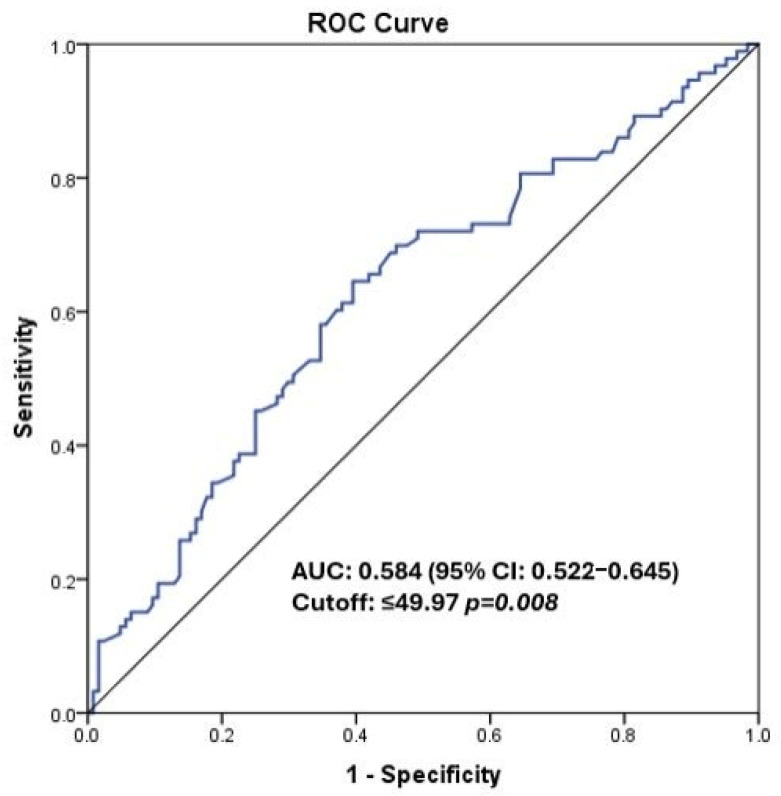
ROC curve for PNI (AUC: 0.584).

The optimal cut-off value for PNI was determined using receiver operating characteristic (ROC) curve analysis, yielding a threshold of 49.97 (Figure 1). This value was used for patient stratification in all subsequent analyses. The proximity of this threshold to the cohort median (49.1) was noted as a supportive observation.

### 2.4. Statistical Analysis

Statistical analyses were performed using IBM SPSS Statistics, Version 25.0 (IBM Corp., Armonk, NY, USA). Continuous variables were summarized as mean ± standard deviation or median and interquartile range (IQR), according to their distribution, while categorical variables were reported as frequencies and percentages. The distribution of continuous variables was assessed before selecting the appropriate summary measure and comparative test. ROC curve analysis was used to determine the optimal PNI cut-off value for predicting mortality. Survival analyses were performed using the Kaplan–Meier method and compared using the log-rank test. Multivariate Cox regression models were used to identify independent prognostic factors affecting OS and PFS. A *p*-value < 0.05 was considered statistically significant. To account for potential confounding due to baseline imbalances between treatment groups, multivariate Cox proportional hazards regression models were constructed, including clinically relevant variables such as age, ECOG performance status, comorbidity status, T stage, and N stage. No propensity score matching or weighting methods were applied.

## 3. Results

A total of 262 patients were included in this study. The mean age was 66.35 ± 8.92 years, and 84% were male. NAC was administered to 138 patients (52.7%) and AC to 124 (47.3%). Baseline demographic and clinicopathological characteristics according to treatment group are summarized in Table 1. ECOG performance status and comorbidity prevalence differed significantly between treatment groups. Additionally, tumor stage and nodal stage distributions differed between groups. PNI values demonstrated a skewed distribution; therefore, median and interquartile range were used to summarize central tendency.

**Figure 2:**
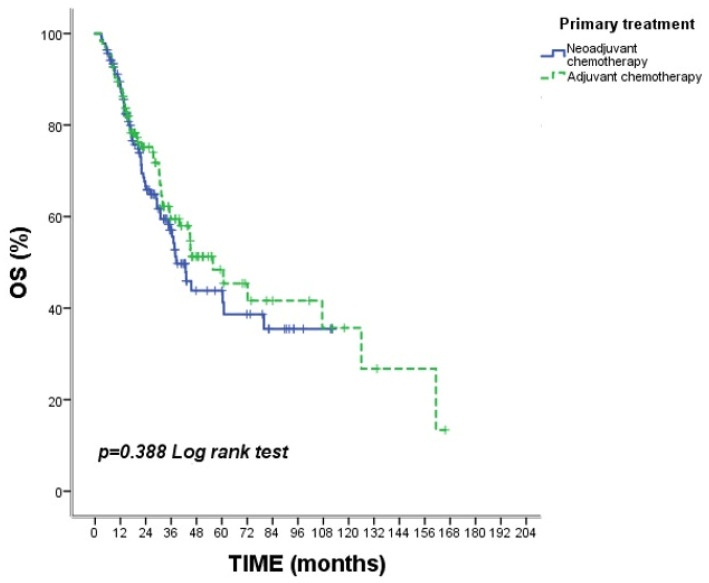
Kaplan–Meier curves for overall survival (OS) according to treatment strategy (neoadjuvant vs. adjuvant chemotherapy). No statistically significant difference in overall survival was observed between the NAC and AC groups (*p* = 0.388). Tick marks indicate censored observations.

**Figure 3:**
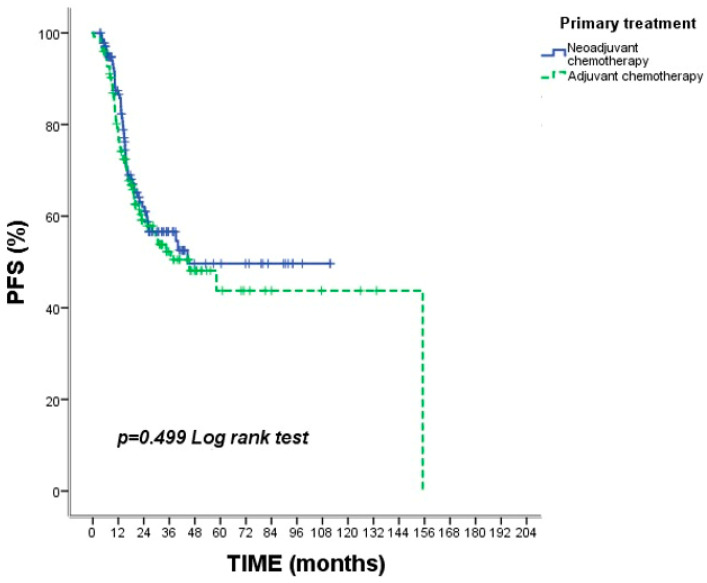
Kaplan–Meier curves for progression-free survival (PFS) according to treatment strategy. Progression-free survival did not differ significantly between treatment groups (*p* = 0.499). Tick marks indicate censored observations.

When OS and PFS were compared between the NAC and AC groups, no statistically significant difference was observed (Table 2). Median OS was 21.1 months (95% CI: 17.1–23.0) in the NAC group and 19.3 months (95% CI: 17.7–26.0) in the AC group (*p* = 0.388). Median PFS was 15.2 months (95% CI: 14.0–18.9) and 15.6 months (95% CI: 12.8–18.9), respectively (*p* = 0.499) (Figure 2 and Figure 3).

**Figure 4:**
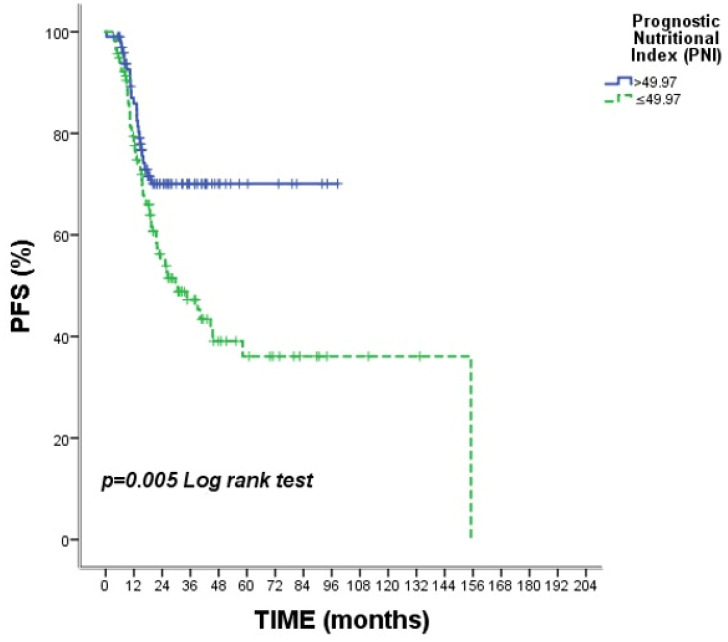
Kaplan–Meier curves for progression-free survival stratified by Prognostic Nutritional Index (PNI). A significant difference in progression-free survival was observed according to PNI status (*p* = 0.005). Tick marks indicate censored observations.

The Kaplan–Meier analysis for progression-free survival is demonstrated in Table 3. Most clinical variables were not significantly associated with PFS. Nodal stage showed a significant association with disease progression (*p* = 0.022), with N0 patients demonstrating the most favorable outcomes compared to N3 patients. Pathological complete response following neoadjuvant chemotherapy was strongly associated with improved PFS (*p* < 0.001). Additionally, PNI was significantly associated with PFS (*p* = 0.005), with patients with PNI >49.97 exhibiting superior outcomes. (Figure 4).

**Figure 5:**
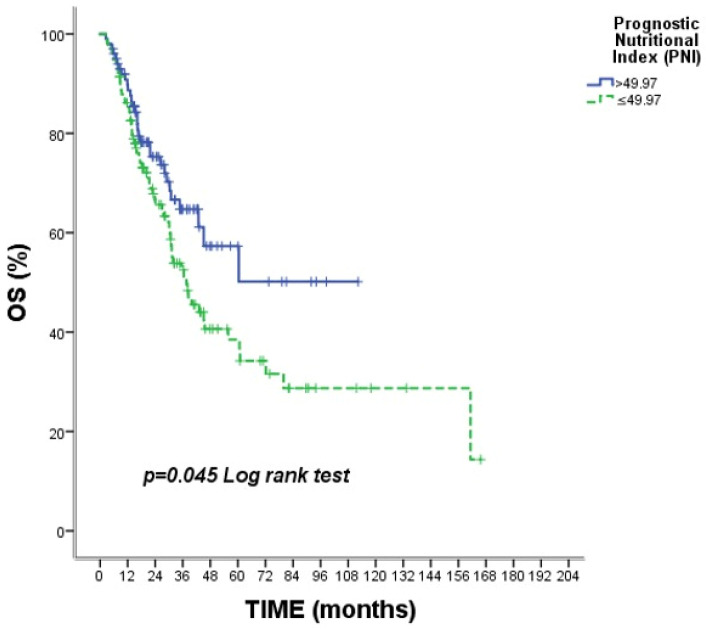
Kaplan–Meier curves for overall survival stratified by PNI. Footnote: Kaplan–Meier curves for overall survival stratified by Prognostic Nutritional Index (PNI). Patients with PNI > 49.97 demonstrated significantly improved overall survival compared with those with lower PNI values (*p* = 0.045). Tick marks indicate censored observations.

Several clinical variables were evaluated for their association with overall survival (Table 4). Nodal stage showed a significant association with survival (*p* = 0.034), with N0 patients demonstrating markedly better survival than N3 patients. Among patients receiving neoadjuvant chemotherapy, pCR was significantly associated with improved survival (*p* = 0.008). Additionally, PNI demonstrated a significant association with overall survival (*p* = 0.045), with patients with PNI >49.97 exhibiting superior survival outcomes. A correlation analysis between PNI and CRP levels showed no statistically significant association (r = 0.102, *p* = 0.467). These findings suggest that nodal stage, pCR, and PNI are important prognostic indicators for overall survival in this patient population. (Figure 5).

Multivariate Cox regression analysis was performed to identify independent prognostic factors for PFS (Table 5). Nodal stage was an independent predictor of disease progression (*p* = 0.049). In particular, patients with N3 disease had a significantly increased risk of progression compared to N0 patients (HR = 5.92, 95% CI: 1.06–32.84; *p* = 0.042). The absence of pCR following neoadjuvant chemotherapy showed a trend toward increased progression risk (HR = 2.15, 95% CI: 0.83–5.55), but this did not reach statistical significance (*p* = 0.113). Similarly, PNI did not retain independent prognostic significance in multivariate analysis (HR = 1.61, 95% CI: 0.80–3.24; *p* = 0.175). Treatment type was also not significantly associated with PFS (*p* = 0.558). Overall, advanced nodal stage was the only independent predictor of disease progression in this cohort.

Multivariate Cox regression analysis was performed to identify independent prognostic factors for OS (Table 6). Although nodal stage demonstrated a trend toward worse survival with increasing stage, this did not reach statistical significance in multivariate analysis (*p* = 0.185). Similarly, the absence of pCR following neoadjuvant chemotherapy was associated with an increased risk of death (HR = 1.66, 95% CI: 0.70–3.96), but did not reach statistical significance (*p* = 0.247). Importantly, PNI remained an independent prognostic factor for overall survival; patients with PNI ≤ 49.97 had a significantly higher risk of mortality compared with those with PNI > 49.97 (HR = 1.78, 95% CI: 1.04–3.38; *p* = 0.044). Treatment type was not significantly associated with OS (*p* = 0.602). These findings highlight the independent prognostic role of nutritional and immunological status in patients with locally advanced bladder cancer.

## 4. Discussion

This multicenter real-world study evaluated survival outcomes and prognostic factors in patients with locally advanced bladder cancer receiving perioperative chemotherapy. This study represents one of the few multicenter analyses evaluating both treatment sequencing and host-related inflammatory biomarkers in this patient population. The main findings can be summarized as follows: treatment sequencing between NAC and AC did not significantly affect survival; nodal stage emerged as a key determinant of disease progression; and PNI was an independent prognostic factor for overall survival. These findings suggest that tumor biology and host-related systemic factors may play a more prominent role in determining prognosis than treatment sequencing alone.

Cisplatin-based NAC followed by radical cystectomy is currently considered the standard of care for eligible patients with muscle-invasive bladder cancer, based on randomized evidence demonstrating improved survival compared with surgery alone [3,4]. In our study, however, survival outcomes were comparable between the NAC and AC groups. Median OS was 21.1 months in the NAC group and 19.3 months in the AC group (*p* = 0.388), while median PFS was 15.2 and 15.6 months, respectively (*p* = 0.499). These median OS values are broadly comparable to those reported in real-world cohorts of locally advanced bladder cancer receiving perioperative chemotherapy, where median OS typically ranges from 18 to 26 months depending on disease stage and patient selection [18,19]. A large SEER-based cohort study by Tan et al. reported no significant difference in OS or cancer-specific survival between NAC and AC after propensity score adjustment [2]. Similarly, analyses from the RISC database and other national registry studies have suggested comparable survival outcomes between treatment strategies in real-world settings [18,19]. It is important to note that patients in our AC group tended to present with more advanced tumor and nodal stages, reflecting the inherent selection bias of retrospective analyses and likely explaining the absence of a survival advantage for NAC. These findings highlight the importance of baseline disease characteristics when interpreting real-world treatment comparisons, and reinforce that the delivery of perioperative chemotherapy itself—regardless of sequencing—may be the critical determinant of outcome [6,7]. Although multivariate adjustment was performed to account for baseline differences, such approaches may not fully eliminate selection bias inherent to retrospective analyses. The absence of propensity score-based methods represents an additional limitation, and residual confounding cannot be excluded. Similar methodological concerns have been highlighted in previous studies evaluating perioperative chemotherapy strategies in bladder cancer, emphasizing the importance of careful adjustment for baseline differences [20]. Therefore, comparisons between treatment groups should be interpreted with caution, and future studies incorporating propensity score-based methods may provide more robust estimates. Of note, the recent NIAGARA phase 3 trial demonstrated that adding perioperative durvalumab to NAC significantly improved event-free survival and overall survival compared with NAC alone, suggesting that the future perioperative standard of care may shift toward chemoimmunotherapy combinations [5].

Pathological complete response following NAC has consistently been identified as one of the strongest predictors of favorable survival in bladder cancer [9,10]. Reported pCR rates with cisplatin-based NAC range from 20% to 40%, and patients achieving pCR demonstrate substantially improved long-term outcomes, with some series reporting 5-year OS rates exceeding 80% [9,21]. In our cohort, pCR was strongly associated with improved OS and PFS in univariate analyses (*p* = 0.008 and *p* < 0.001, respectively). However, pCR did not retain independent significance in multivariate analysis. This finding may reflect several factors: first, the strong collinearity between pCR and nodal downstaging in our cohort; second, patients achieving pCR represented a relatively small subset, potentially limiting statistical power in the multivariate model; and third, it is recognized that pCR does not eliminate recurrence risk, particularly in node-positive patients [21]. Despite these limitations, the strong univariate association supports the growing interest in response-adapted treatment strategies and highlights the importance of achieving maximal tumor downstaging during NAC.

Lymph node involvement remains one of the most powerful prognostic determinants in bladder cancer. Numerous studies have demonstrated that nodal metastasis is independently associated with decreased survival and increased recurrence risk following radical cystectomy [11,12]. In our study, nodal stage was significantly associated with both OS (*p* = 0.034) and PFS (*p* = 0.022) in univariate analyses. Importantly, N3 disease independently predicted disease progression in multivariate analysis with an approximately six-fold increase in risk compared with N0 patients (HR 5.92; *p* = 0.042). These findings are consistent with data from Wagner et al., who reported both radiological and pathological nodal status as independent survival predictors in patients with locally advanced bladder cancer undergoing NAC and cystectomy [22]. The aggressive biological behavior associated with extensive nodal involvement underscores the need for accurate preoperative staging and individualized systemic treatment planning in node-positive patients.

One of the most notable findings of our study is the independent prognostic significance of PNI for overall survival. Cancer-related systemic inflammation and nutritional depletion are increasingly recognized as modulators of tumor progression and treatment response [13,14]. PNI simultaneously reflects immune competence and nutritional status through a simple calculation using routinely available laboratory parameters. Previous meta-analyses have demonstrated that low PNI is associated with poor OS in bladder cancer, with pooled hazard ratios ranging from 1.67 to 1.78 [17,23]. A 2024 systematic review and meta-analysis including 2951 bladder cancer patients confirmed a significant association between low PNI and poor prognosis across multiple disease settings [24]. In our study, patients with PNI ≤ 49.97 had a significantly higher risk of mortality in multivariate analysis (HR 1.78, 95% CI 1.04–3.38; *p* = 0.044), independent of tumor stage and treatment strategy. The ROC-derived cut-off of 49.97 was closely aligned with the cohort median (49.1), consistent with values reported across prior studies where PNI thresholds have ranged from 44.7 to 52.6 [16,17,23]. The moderate discriminatory performance of PNI (AUC 0.584) in our cohort is consistent with prior studies, suggesting that PNI functions best as a complementary rather than a standalone prognostic marker [16,17]. Although PNI is considered a marker reflecting both nutritional and inflammatory status, we did not observe a significant correlation with CRP in our cohort. This may suggest that PNI in this population is more reflective of overall host condition than of acute inflammatory burden; however, CRP alone may not fully reflect systemic inflammation. The selection of the PNI cut-off remains a methodological challenge, as different thresholds have been used across studies. The ROC-derived cut-off was used consistently throughout the analyses, and its proximity to the cohort median supports its representativeness. Moreover, the modest AUC value indicates that PNI should be interpreted as a supportive rather than a standalone prognostic marker.

Despite this, PNI is inexpensive, non-invasive, and can be calculated from routine pretreatment blood work. Patients identified as having low PNI may represent a high-risk subgroup warranting preoperative nutritional optimization, more intensive surveillance, or consideration of additional systemic support strategies to improve treatment tolerance and outcomes [24,25].

Several limitations of this study should be acknowledged. The retrospective design introduces potential selection bias, and treatment regimens may have varied across participating centers. Although the sample size is relatively large for a real-world cohort, certain subgroup analyses—particularly those involving N3 disease and pCR—may be underpowered. Additionally, PNI is a dynamic marker, and pretreatment values may not fully capture changes in nutritional and immune status during or after chemotherapy. The lack of detailed data on individual comorbidities and infection status, including urinary tract infections, represents an additional limitation, as these factors may influence PNI values.

Despite these limitations, the multicenter design strengthens the generalizability of our findings and reflects the diversity of real-world clinical practice.

## 5. Conclusions

In this multicenter real-world cohort, nodal stage and systemic inflammatory-nutritional status were the key determinants of prognosis in patients with locally advanced bladder cancer receiving perioperative chemotherapy. The Prognostic Nutritional Index emerged as an independent predictor of overall survival, supporting its potential role in pretreatment risk stratification. Treatment sequencing between neoadjuvant and adjuvant chemotherapy was not associated with significant differences in survival outcomes; however, these findings should be interpreted with caution due to potential residual confounding. Future prospective studies are warranted to validate these findings and to evaluate the clinical utility of nutritional and inflammatory biomarkers in guiding individualized treatment decisions in this patient population.

## Figures and Tables

**Table 1:** Baseline Demographic and Clinicopathological Characteristics According to Treatment Group.

	Total n = 262	NAC n = 138	AC n = 124	*p*
Age (Mean ± SD)	66.35 ± 8.92	65.53 ± 9.60	67.26 ± 8.05	0.118 ^a^
Sex				
Male	220 (84.0)	118 (85.5)	102 (82.3)	0.474 ^b^
Female	42 (16.0)	20 (14.5)	22 (17.7)	
ECOG-PS				
0	121 (46.2)	75 (54.3)	46 (37.1)	0.004 ^b^
1	124 (47.3)	59 (42.8)	65 (52.4)	
2	17 (6.5)	4 (2.9)	13 (10.5)	
Comorbidity				
No	111 (42.4)	70 (50.7)	41 (33.1)	0.004 ^b^
Yes	151 (57.6)	68 (49.3)	83 (66.9)	
Grade				
1	25 (10.9)	15 (12.5)	10 (9.1)	0.678 ^b^
2	49 (21.3)	26 (21.7)	23 (20.9)	
3	156 (67.8)	79 (65.8)	77 (70)	
T stage				
T1	11 (4.2)	9 (6.5)	2 (1.6)	<0.001 ^b^
T2	115 (44.2)	84 (60.9)	31 (25.4)	
T3	95 (36.5)	38 (27.5)	57 (46.7)	
T4	39 (15.0)	7 (5.1)	32 (26.2)	
N stage				
N0	135 (51.5)	84 (60.9)	51 (41.1)	0.002 ^b^
N1	66 (25.2)	33 (23.9)	33 (26.6)	
N2	52 (19.8)	16 (11.6)	36 (29)	
N3	9 (3.4)	5 (3.6)	4 (3.2)	
Stage				
1	26 (9.9)	9 (6.5)	1 (0.8)	<0.001 ^b^
2	81 (30.9)	61 (44.2)	20 (16.1)	
3	155 (59.2)	68 (49.3)	87 (70.2)	
PNI (Median (IQR))	50.0 (10.7)	51.45 (11.34)	47.47 (9.97)	0.331 ^a^

Abbreviations: NAC: neoadjuvant chemotherapy; AC: adjuvant chemotherapy; OS: overall survival; PFS: progression-free survival; ECOG-PS: Eastern Cooperative Oncology Group Performance Status; PNI: prognostic nutritional index; CI: confidence interval. ^a^ *p*-values calculated using Student’s *t*-test or Mann–Whitney U test, as appropriate. ^b^ *p*-values calculated using the Chi-square test or Fisher’s exact test, as appropriate.

**Table 2:** Survival outcomes according to treatment strategy.

		NAC (n = 138)	AC (n = 124)	*p*
PFS	Median (months)	15.2	15.6	0.499
	95% CI	14.0–18.9	12.8–18.9	
OS	Median (months)	21.1	19.3	0.388
	95% CI	17.1–23.0	17.7–26.0	

Abbreviations: NAC: neoadjuvant chemotherapy; AC: adjuvant chemotherapy; OS: overall survival; PFS: progression-free survival; CI: confidence interval.

**Table 3:** Progression-free survival according to clinical variables.

	2-Year PFS (%)	5-Year PFS (%)	Median PFS (Months) (95% CI)	*p*
Overall	60.7	49.0	45.50 (28.15–62.85)	
Age				
<65	58.2	42.5	34.50 (21.94–47.05)	0.258
>65	61.9	49.3	58.20 (-)	
Sex				
Male	62.0	45.4	44.56 (26.02–63.11)	0.597
Female	53.5	53.5	- (-)	
T stage				
T1	54.5	36.4	24.60 (13.66–35.53)	0.134
T2	63.0	46.3	58.20 (-)	
T3	65.6	55.2	- (-)	
T4	47.2	35.8	18.66 (3.92–34.40)	
N stage				
N0	68.3	60.4	100.40 (-)	0.022
N1	55.3	35.2	30.60 (9.63–51.56)	
N2	54.2	38.5	26.36 (14.91–37.81)	
N3	40.0	20.0	15.46 (8.52–22.40)	
Stage				
Stage-1	76.2	76.2	- (-)	
Stage-2	63.5	48.0	44.56 (-)	
Stage-3	56.6	40.7	34.50 (18.81–50.18)	
Treatment type				
NAC	62.0	49.6	44.56 (-)	0.499
AC	59.1	43.7	45.50 (18.59–74.40)	
pCR				
Yes	85.9	80.0	- (-)	<0.001
No	50.9	37.2	24.40 (4.85–43.94)	
PNI				
>49.97	70.1	70.1	- (-)	0.005
≤49.97	55.0	36.1	30.60 (16.10–45.09)	

Abbreviations: NAC: neoadjuvant chemotherapy; AC: adjuvant chemotherapy; pCR: pathological complete response; PNI: prognostic nutritional index; CI: confidence interval. (-) indicates non-estimable median survival or confidence interval values due to insufficient events during follow-up.

**Table 4:** Overall survival according to clinical variables.

	2-Year (%)	5-Year (%)	Median (Months) (95% CI)	*p*
Overall	68.6	48.5	47.63 (34.90–60.35)	
Age				
<65	72.0	48.0	47.80 (26.32–69.27)	0.719
>65	66.8	48.4	45.53 (27.92–63.14)	
Sex				
Male	68.7	45.4	45.10 (31.44–58.75)	0.094
Female	69.2	69.2	- (-)	
T stage				
T1	72.7	43.6	63.00 (0.61–125.38)	0.143
T2	75.8	52.2	60.90 (28.47–93.32)	
T3	62.8	46.2	45.00 (25.90–64.09)	
T4	63.7	34.3	35.26 (24.65–45.87)	
N stage				
N0	74.1	63.0	107.40 (43.26–171.53)	0.034
N1	76.1	44.0	42.86 (33.97–51.75)	
N2	63.9	34.0	37.63 (32.04–43.21)	
N3	51.5	36.4	24.43 (4.38–44.48)	
Stage				
1	82.7	82.7	- (-)	
2	68.2	54.2	64.13 (32.40–95.86)	
3	69.2	40.6	38.46 (30.50–46.42)	
Treatment type				
NAC	66.7	43.8	38.46 (30.70–46.22)	0.388
AC	75.2	48.4	55.76 (34.05–77.48)	
pCR				
Yes	81.4	55.2	- (-)	0.008
No	57.8	37.6	30.90 (14.19–47.60)	
PNI				
>49.97	75.3	57.3	- (-)	0.045
≤49.97	66.7	38.5	37.63 (26.70–48.55)	

NAC: neoadjuvant chemotherapy; AC: adjuvant chemotherapy; pCR: pathological complete response; PNI: prognostic nutritional index; CI: confidence interval. (-) indicates non-estimable median survival or confidence interval values due to insufficient events during follow-up.

**Table 5:** Multivariate Cox regression analysis for PFS.

		HR (%95 CI)	*p*
N stage	N0	ref	
	N1	0.75 (0.34–1.67)	0.489
	N2	1.55 (0.72–3.36)	0.258
	N3	5.92 (1.06–32.84)	0.042
pCR	Yes	ref	0.113
	No	2.15 (0.83–5.55)	
PNI	>49.97	ref	0.175
	≤49.97	1.61 (0.80–3.24)	
Treatment type	AC	ref	
	NAC	1.09 (0.82–1.46)	0.558

NAC: neoadjuvant chemotherapy; AC: adjuvant chemotherapy; pCR: pathological complete response; PNI: prognostic nutritional index; CI: confidence interval; ref: reference category.

**Table 6:** Multivariate Cox regression analysis for overall survival.

		HR (%95 CI)	*p*
N stage	N0	ref	
	N1	0.83 (0.41–1.68)	0.608
	N2	1.39 (0.68–2.82)	0.357
	N3	4.18 (0.79–21.93)	0.091
pCR	Yes	ref	0.247
	No	1.66 (0.70–3.96)	
PNI	>49.97	ref	0.044
	≤49.97	1.78 (1.04–3.38)	
Treatment type	AC	ref	
	NAC	0.91 (0.65–1.28)	0.602

NAC: neoadjuvant chemotherapy; AC: adjuvant chemotherapy; pCR: pathological complete response; PNI: prognostic nutritional index; CI: confidence interval; ref: reference category.

## Data Availability

The datasets are available from the corresponding author on reasonable request.
